# The profile inter‐unit reliability

**DOI:** 10.1111/biom.13167

**Published:** 2019-11-10

**Authors:** Kevin He, Claudia Dahlerus, Lu Xia, Yanming Li, John D. Kalbfleisch

**Affiliations:** ^1^ Department of Biostatistics University of Michigan Ann Arbor Michigan; ^2^ School of Public Health, Kidney Epidemiology and Cost Center University of Michigan Ann Arbor Michigan

**Keywords:** health provider profiling, inter‐unit reliability, national dialysis data, quality of care, reliability

## Abstract

To assess the quality of health care, patient outcomes associated with medical providers (eg, dialysis facilities) are routinely monitored in order to identify poor (or excellent) provider performance. Given the high stakes of such evaluations for payment as well as public reporting of quality, it is important to assess the reliability of quality measures. A commonly used metric is the inter‐unit reliability (IUR), which is the proportion of variation in the measure that comes from inter‐provider differences. Despite its wide use, however, the size of the IUR has little to do with the usefulness of the measure for profiling extreme outcomes. A large IUR can signal the need for further risk adjustment to account for differences between patients treated by different providers, while even measures with an IUR close to zero can be useful for identifying extreme providers. To address these limitations, we propose an alternative measure of reliability, which assesses more directly the value of a quality measure in identifying (or profiling) providers with extreme outcomes. The resulting metric reflects the extent to which the profiling status is consistent over repeated measurements. We use national dialysis data to examine this approach on various measures of dialysis facilities.

## 1 INTRODUCTION

Monitoring outcomes of health care providers is an important activity that has received much attention in the literature (eg, Normand *et al*., 1997; Normand and Shahian, 2007; Jones and Spiegelhalter, 2011; He *et al*., 2013; Estes *et al*., 2018). In order to identify extreme (poor or excellent) performance and to intervene as necessary, outcomes of patients associated with health care providers are routinely monitored most often by both government and private payers. This monitoring can help patients make more informed decisions and can also aid consumers, stakeholders, and payers in identifying providers where improvement may be needed, and even closing or fining those with extremely poor outcomes. Therefore, it is important that the quality measures used for profiling providers are appropriate and one aspect of this is the measure's reliability.

To assess the reliability of a quality measure, the inter‐unit reliability (IUR) is commonly used. The IUR specifies the proportion of the total variation in the quality measure that can be attributed to the between‐provider variation. The variation in a specific measure across health care providers can be viewed as comprising two parts: the between‐provider variation and the within‐provider variation. The IUR is then defined as the ratio of the between‐provider variance to the total variance.

Scholle *et al*. (2008) and Adams (2009) suggested that a quality measure should attain an IUR of at least 0.7. This work has recently been discussed by the National Quality Forum as suggesting a possible guideline for assessing measure reliability. If the IUR is large, it is argued that most of the variation observed between health care providers is driven by systematic differences between the providers and not by the variation in the outcomes of the patients being treated. Thus, it is argued that the measure gives a reliable assessment of between‐provider differences and could be used for the purpose of assessing extreme providers. Note, however, that Kalbfleisch *et al*. (2018) discussed several drawbacks of using the IUR to characterize the suitability of a measure for profiling providers. In particular,

1. The variation between providers may be due to various factors in addition to differences in the quality of the health care provided. Differences between providers can also arise because there are important unmeasured characteristics, such as patient comorbidities or patient and provider demographics, that are not within the control of the provider and that differ across providers. Thus, a large IUR can be a signal of incomplete risk adjustment and may not be much related to the quality of care at all.
2. The IUR may not determine the suitability of a measure for identifying outliers. Even measures with an IUR close to zero can be very useful for identifying extreme providers, whose outcomes do not conform to an assumed statistical model.

In this paper, we propose an additional metric of reliability that assesses more directly the value of a quality measure in identifying providers with extreme outcomes. The underlying idea is that we should consider a measure to be reliable if, on repeated applications, it profiles the same providers as being extreme with relatively high probability. We proceed in two steps: first, we evaluate the ability of a measure to consistently profile providers with extreme outcomes; second, we use the IUR to calibrate this new metric, which we call the profile IUR (termed PIUR throughout this paper).

Our paper continues as follows: Section 2 first reviews the IUR for a simple linear model and then defines the proposed PIUR for normally distributed patient outcomes. Section 3 exemplifies the PIUR for several commonly used profiling methods. In Sections 4 and 5, we examine the proposed PIUR with simulations and national data on dialysis patients. We conclude with a discussion in Section 6.

## 2 PROFILE IUR

### 2.1 A simple linear model and review of the IUR

Let $Y_{ij}^{*}$ represent a continuous outcome for subject j in provider i, where i=1,…,m and $j=1,\text{…},n_{i}$. Here m is the total number of providers and $n_{i}$ is the sample size for provider i. Consider an underlying linear regression model

(1) $$Y_{ij}^{*}=\mu +\alpha_{i}+X_{ij}^{T}\beta +\epsilon_{ij},$$

where $\alpha_{i}∼N\left(0,\sigma_{b}^{2}\right)$ is the provider effect, $\epsilon_{ij}∼N\left(0,\sigma_{w}^{2}\right)$ is the random noise, and $X_{ij}$ is a vector of patient characteristics. The regression coefficients, β, measure the within‐provider relationship between the covariates and the response. Here we assume that large values of $Y_{ij}^{*}$ correspond to poor outcomes.

In model (1), it is common to assume (at least implicitly) that $X_{ij}$ is independent of $\alpha_{i}$. However, in practice, patient characteristics can be correlated with provider attributes (eg, patients with less favorable health status may be referred to providers with poorer treatment strategies). In this case, the estimated regression coefficients based on the usual likelihood analysis of the model (1) are biased. Alternatively, β can be estimated in a model with fixed effects for provider‐specific parameters, which avoids the aforementioned issues of bias (Kalbfleisch and Wolfe, 2013). The resulting estimate can then be used as an offset to estimate the remaining parameters and the $\alpha_{i}$'s.

We note that, in many profiling applications, the number of providers and the number of patients are large so that $\mu ,\sigma_{b},\sigma_{w}$, and β can be precisely estimated. To simplify the notation, we proceed below as though their values are known. Let $Y_{ij}=Y_{ij}^{*}-\mu -X_{ij}^{T}\beta$ be the risk‐adjusted response, so that the model (1) becomes

(2) $$Y_{ij}=\alpha_{i}+\epsilon_{ij}.$$

An estimate of $\alpha_{i}$ is ${\bar{Y}}_{i}=\sum_{j=1}^{n_{i}}Y_{ij}∕n_{i}$, where ${\bar{Y}}_{i}∼N\left(0,\sigma_{b}^{2}+\sigma_{w}^{2}∕n_{i}\right)$. Here $\sigma_{b}^{2}$ is the between‐provider variance, and $\sigma_{w}^{2}∕n_{i}$ is the within‐provider variance.

The IUR for a provider with $n_{i}$ patients is the proportion of the total variation in ${\bar{Y}}_{i}$ that can be attributed to the between‐provider variation:

$${\;\text{IUR}\;}_{i}=\frac{\sigma_{b}^{2}}{\sigma_{b}^{2}+\sigma_{w}^{2}∕n_{i}},$$

which is also the square of the correlation between ${\bar{Y}}_{i}$ and the true provider effect $\alpha_{i}$

$${\;\text{IUR}}_{i}={\text{Corr}\;}^{2}\left({\bar{Y}}_{i},\alpha_{i}\right).$$

**Figure 1:**
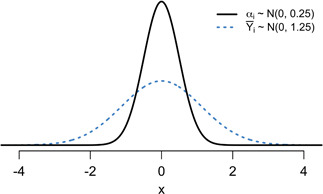
The solid line is the distribution of the true provider effect, $\alpha_{i}$, and the dotted line is the distribution of the estimated provider effect, ${\bar{Y}}_{i}$, in the example with $\sigma_{w}=10,\sigma_{b}=0.5$, and $n_{i}=100$. The IUR compares the variance of the former distribution to that of the latter [This figure appears in color in the electronic version of this article, and any mention of color refers to that version]

Figure 1 plots the density of an example with two distributions of interest: the distribution of the provider effects, $\alpha_{i}$, and the distribution of the estimated provider effects, ${\bar{Y}}_{i}$. The IUR is the ratio of the variances of these two distributions.

To assess the average ability of a quality measure to distinguish between providers, the overall IUR can be obtained from the decomposition of total variation in a one‐way analysis of variance. Recall that the between sums of squares (SSB) is defined as $SSB=\sum_{i=1}^{m}n_{i}{\left(\bar{Y_{i}}-\bar{\bar{Y}}\right)}^{2}$ with $\bar{\bar{Y}}=\sum_{i=1}^{m}\sum_{j=1}^{n_{i}}Y_{ij}∕\sum_{i=1}^{m}n_{i}$ and $E\left(SSB\right)=\left(m-1\right)\left(\sigma_{w}^{2}+n\prime \sigma_{b}^{2}\right)$, where

$$n\prime =\frac{1}{m-1}\left(\sum_{i=1}^{m}n_{i}-\frac{\sum_{i=1}^{m}n_{i}^{2}}{\sum_{i=1}^{m}n_{i}}\right).$$

The overall IUR across providers is then defined with respect to the average provider size n′

$$IUR=\frac{\sigma_{b}^{2}}{\sigma_{b}^{2}+\sigma_{w}^{2}∕n\prime }.$$

### 2.2 Limitations of the IUR

The interpretation of the IUR depends on the sources of variation in the provider effects. The argument that a relatively large value of the IUR is required to justify the use of a measure for profiling is based on the assumption that the variation in the provider effects is entirely due to the quality of care (Adams, 2009; Kalbfleisch *et al*., 2018). That is, there are no unobserved confounders that are correlated with the outcome of interest and whose values vary across providers. This assumption, however, is often invalid. For example, unobserved socio‐economic factors, comorbidities, and genetic differences of patients may differ substantially across providers and so contribute to the between‐provider variation. Thus, a large IUR can be a signal of incomplete risk adjustment. Moreover, the IUR indicates the average ability of the measure to distinguish between providers. In identifying providers that are outliers, however, we are not concerned with this average, but rather with the measure's ability to identify providers where outcomes are extreme. Thus, a measure with a small IUR may still be useful in identifying a few providers whose outcomes are extreme. These concerns motivate us to propose an alternative measure of reliability, which emphasizes upon the identification of outliers.

### 2.3 A model with outliers and the PIUR

It is convenient to extend the model (1) to include contamination with outliers. Such a model is considered by Efron (2013) for large‐scale hypothesis testing. Suppose that the provider effects are either from the null normal distribution, $N\left(0,\sigma_{b}^{2}\right)$ with probability $\pi_{0}$ or from a distribution of outliers with probability $\pi_{1}=1-\pi_{0}$. Thus, we generalize model (1) by taking

(3) $$f\left(\alpha_{i}\right)=\pi_{0}f_{0}\left(\alpha_{i}\right)+\pi_{1}f_{1}\left(\alpha_{i}\right),$$

where $f_{0}\left(\alpha \right)$ is the $N\left(0,\sigma_{b}^{2}\right)$ density and $f_{1}\left(\alpha \right)$ is a density for outliers with support on the region α>C for some specified C>0, say $C=2\sigma_{b}$.

For provider profiling, it is natural to consider a quality measure as reliable if it is able to reliably identify the same providers as extreme. Thus, we might assess a measure by its propensity to identify the same providers as extreme when the data are replicated. Replication is not possible in practice, so we consider a sample‐splitting approach as follows. Randomly divide each providers patients into two nearly equal‐sized subgroups. For a given threshold, determine whether each provider is identified as extreme in the first and the second subgroups. Repeat this process a large number of times to estimate the empirical probability that a provider is profiled in the second subgroup given that it is profiled in the first. This empirical reflagging rate is then put on the IUR scale, by determining the IUR that would yield this reflagging rate in the absence of outliers. The difference between the PIUR and the IUR indicates the extent to which the measure identifies outliers.

More specifically, given a data set, a quality measure and a profiling method, PIUR is determined as follows:

Algorithm 1 (*Definition of the PIUR*)

1. Randomly divide each provider's patients into two nearly equal‐sized subgroups, for example, groups A and B.
2. For a given threshold p and profiling method, determine whether the provider is flagged based on data in groups A and B.
3. Repeat this process a large number of times, and estimate the probability that a provider is flagged in group B given that it is flagged in group A. This estimate is the empirical reflagging rate ${\hat{\theta }}_{B|A}$.
4. Under the assumption that the data arise from the model (2) with no outliers, let G(R) = *Pr*(provider is flagged in group B| provider is flagged in group A, IUR = R).
5. The PIUR at level p is $\tilde{R}$, where $G\left(\tilde{R}\right)={\hat{\theta }}_{B|A}$.

As defined, the PIUR is on the same scale as the IUR, but with emphasis on the ability of quality measures to consistently identify outliers. For example, for a given empirical reflagging rate ${\hat{\theta }}_{B|A}$, we solve the equation $G\left(R\right)={\hat{\theta }}_{B|A}$ and find the R that leads to the empirical reflagging rate ${\hat{\theta }}_{B|A}$. The values of the PIUR, compared with the IUR, are influenced by the proportion of outliers and their magnitude. That is, a higher PIUR compared to the IUR indicates the presence of outlier providers, which is not captured in the IUR itself.

## 3 PROFILING METHODS

In this section, we briefly review several commonly used profiling methods for flagging extreme providers. We show that considering the provider‐specific IUR provides a simple theoretical justification for estimating the proposed PIUR for various profiling methods. That is, assuming that the data arise from the model (2), the conditional probability for the *i*th provider, $G_{i}\left(R\right)$, depends only on the ${\text{IUR}}_{i}$.

### 3.1 Provider effects due entirely to variation in the quality of care

In these cases, it is natural to consider tests of sharp null hypotheses about the provider effects. Generally one of two methods is used: fixed effects (FE) and random effects (RE).

Under the linear model, the fixed effects *Z*‐score for a test of $\alpha_{i}=0$ is

$$Z_{FE,i}={\bar{Y}}_{i}∕\left(\sigma_{w}∕\sqrt{n_{i}}\right).$$

Based on fixed effects, the ith provider is flagged as worse than expected if $Z_{FE,i}>z_{p}$, where $z_{p}$ is the upper *P*th quantile of the standard normal distribution, say for P=.05 or .025.

Let $Z_{FE,i}^{\left(A\right)}$ and $Z_{FE,i}^{\left(B\right)}$ be the FE‐based *Z*‐scores for the randomly chosen groups A and B within provider i. For a given ${\text{IUR}}_{i}=R$, $Z_{FE,i}^{\left(A\right)}$ and $Z_{FE,i}^{\left(B\right)}$ are bivariate normal with variance 1∕(1−ρ) and correlation ρ=R∕(2−R). The corresponding conditional probability is summarized in Proposition 1.

Proposition 1 Under the linear model (2) with ${\;\text{IUR}\;}_{i}=R$ and p∈(0,1),

$$G_{FE,i}\left(R\right)=Pr\left(Z_{FE,i}^{\left(B\right)}>z_{p}|Z_{FE,i}^{\left(A\right)}>z_{p},{\text{IUR}\;}_{i}=R\right)=\frac{\Phi_{2,\rho }\left(s_{1},s_{1}\right)}{\Phi \left(s_{1}\right)},$$

where $s_{1}=-z_{p}\sqrt{1-\rho }$, Φ and $\Phi_{2,\rho }$ are the cumulative distribution functions of the standard normal distribution and a bivariate normal distribution with variation 1 and correlation ρ, respectively; for example,

$$\Phi_{2,\rho }\left(s_{1},s_{1}\right)=\int_{-\infty }^{s_{1}}\int_{-\infty }^{s_{1}}\frac{1}{2\pi \sqrt{1-\rho^{2}}}\times \text{exp}\left\{-\frac{u^{2}-2\rho uv+v^{2}}{2\left(1-\rho^{2}\right)}\right\}dudv.$$

Alternatively, the RE approach is based on the best linear unbiased predictor (BLUP) or empirical Bayes estimate arising from the “posterior” distribution of $\alpha_{i}$ given the data. The estimate of $\alpha_{i}$ then is ${\hat{\alpha }}_{RE,i}={\;\text{IUR}\;}_{i}{\bar{Y}}_{i}$, which has a posterior variance ${\;\text{IUR}\;}_{i}\sigma_{w}^{2}∕n_{i}$. Thus, the corresponding RE‐based *z*‐score is then given by

$$Z_{RE,i}=\sqrt{{\;\text{IUR}\;}_{i}}Z_{FE,i}.$$

Here ${\;\text{IUR}\;}_{i}$ plays the role of a shrinkage factor.

Let $Z_{RE,i}^{\left(A\right)}$ and $Z_{RE,i}^{\left(B\right)}$ be the RE‐based *Z*‐scores for groups *A* and *B* within provider i. Note that $Z_{RE,i}^{\left(A\right)}$ and $Z_{RE,i}^{\left(B\right)}$ are bivariate normal with variance ρ∕(1−ρ) and correlation ρ.

Proposition 2 Under the linear model (2) with ${\;\text{IUR}\;}_{i}=R$, for a fixed p∈(0,1),

$$G_{RE,i}\left(R\right)=Pr\left(Z_{RE,i}^{\left(B\right)}>z_{p}|Z_{RE,i}^{\left(A\right)}>z_{p},{\text{IUR}\;}_{i}=R\right)=\frac{\Phi_{2,\rho }\left(s_{2},s_{2}\right)}{\Phi \left(s_{2}\right)},$$

where $s_{2}=s_{1}/\sqrt{\rho }$, and ρ is the same as in Proposition 1.

### 3.2 Provider effects are due to incomplete risk adjustment

If quality of care is not the main source of variation in the provider effects, the random variation accounted for in $\sigma_{b}$ should be incorporated in the profiling method. The approach based on fixed effects with random intercept (FERE) (Jones and Spiegelhalter, 2011; Kalbfleisch *et al*., 2018) utilizes fixed effects estimates but judges their values with reference to the marginal distribution, including the between‐provider variation. The *Z*‐score can be constructed as

$$Z_{FERE,i}=\sqrt{1-{\;\text{IUR}\;}_{i}}Z_{FE,i}=\frac{{\bar{Y}}_{i}}{\sqrt{\sigma_{b}^{2}+\sigma_{w}^{2}∕n_{i}}}.$$

One may flag provider i if $Z_{FERE,i}>z_{p}$. This approach is based on the assumption that most of the between‐provider variation is due to unobserved characteristics that are outside the control of the provider. Thus, the FERE approach only flags a provider if its outcome is extreme with reference to the total variation. This approach is useful for identifying providers that are outliers or do not follow the assumed model.

Let $Z_{FERE,i}^{\left(A\right)}$ and $Z_{FERE,i}^{\left(B\right)}$ be the FERE‐based *Z*‐scores for groups A and B in provider i. Note that $Z_{FERE,i}^{\left(A\right)}$ and $Z_{FERE,i}^{\left(B\right)}$ are bivariate normal with variance 1 and covariance ρ.

Proposition 3 Under the linear model (2) with ${\;\text{IUR}\;}_{i}=R$ and p∈(0,1), the conditional probability for the FERE approach is

$$G_{FERE,i}\left(R\right)=Pr\left(Z_{FERE,i}^{\left(B\right)}>z_{p}|Z_{FERE,i}^{\left(A\right)}>z_{p},{\text{IUR}\;}_{i}=R\right)=\frac{\Phi_{2,\rho }\left(s_{3},s_{3}\right)}{\Phi \left(s_{3}\right)},$$

where $s_{3}=-z_{p}$.

Note that Propositions 1 to 3 are based on provider‐specific IUR. To assess the average ability of a quality measure to consistently identify outliers across providers, we extend the PIUR based on the overall IUR. Numerical evaluations for the proposed methods are provided in Section 4.

### 3.3 Empirical null approach

The empirical null approach is based on work of Efron (2004; 2013) who defined the empirical null and used it in problems of assessing false discovery rates. Kalbfleisch and Wolfe (2013) proposed the use of the empirical null in profiling health care providers. We suppose first that all providers are approximately of the same size so that $n_{i}\approx n$ for all i. In the empirical null approach, a normal distribution is fitted to the central part of the distribution of the fixed effects *Z*‐scores, $Z_{FE,i}$, i=1,…,m. This can be done using robust methods that are not influenced by values in the tail of the distribution. For example, one might use *M*‐estimation or maximum likelihood approaches based on a truncated normal model (eg, Efron, 2013). The resulting estimates of the mean and variance are ${\hat{\mu }}_{M}$ and ${\hat{\sigma }}_{M}^{2}$, and the empirical null distribution is $N\left({\hat{\mu }}_{M},{\hat{\sigma }}_{M}^{2}\right)$. This distribution, instead of N(0,1) is used as the null hypothesis with which to assess extreme values of the FE‐*Z*‐scores. More specifically, the ith provider is flagged as worse than expected if $Z_{FE,i}>{\hat{\mu }}_{M}+z_{p}{\hat{\sigma }}_{M}$, where $z_{p}$ is the upper pth quantile of the standard normal distribution.

If the model (2) is exactly true for all providers, the empirical null approach and the FERE approach give essentially the same solution. This follows from the results of Andrews *et al*. (1972) and Huber (1964; 1973), which can be used to show that ${\hat{\mu }}_{M}\to 0$ in probability, and ${\hat{\sigma }}_{M}^{2}$ is a consistent estimate of $\sigma_{b}^{2}+\sigma_{w}^{2}∕n$ as the number of providers m→∞. More generally, however, the empirical null approach also applies to the model (3) where it gives asymptotically correct results, whereas FERE will result in potentially biased estimates of the intercept and $\sigma_{b}^{2}$. As a consequence of the asymptotic equivalence of the empirical null approach and the FERE approach when the model (2) is exactly true, the PIUR of the empirical null approach can be computed by referring the empirical reflagging rate to $G_{FERE}\left(R\right)$. The dependence of the empirical null on sample size can be handled by stratifying the facilities into relatively homogeneous strata as in Kalbfleisch and Wolfe (2013) and He *et al*. (2013). In addition, we have been developing smoothed estimates of the mean and variance of the Z‐scores as a function of sample size so that each provider has an individualized empirical null distribution.

One major advantage of the empirical null approach over FERE or RE is that it generalizes relatively easily to other nonlinear examples where the FE‐based *Z*‐scores are approximately normal for relatively large $n_{i}$. Thus, this approach can be used, for example, in situations where the response is binary as in He *et al*. (2013) and Estes *et al*. (2018) or a failure time as in Kalbfleisch and Wolfe (2013) and in our example in Section 5.

## 4 NUMERICAL EVALUATION

In this section, we examine the properties of the proposed PIUR through numerical evaluation. We consider the FERE‐based *Z*‐scores for an one‐sided test with a significance level *P* = .025. We consider the linear model (2) with $\sigma_{T}^{2}=\sigma_{b}^{2}+\sigma_{w}^{2}∕n\prime =1$. We vary the magnitude of the between‐provider variance, $\sigma_{b}^{2}$, such that ordinary IUR takes values 0.00, 0.25, and 0.50. We assume that the provider effects are either from the null normal distribution $N\left(0,\sigma_{b}^{2}\right)$ with probability $\pi_{0}$, or from a distribution of outliers with probability $\pi_{1}=1-\pi_{0}$. We vary the value of $\pi_{1}$ from 0.00, 0.01, 0.02, and 0.05. The magnitude for these outlier provider effects are fixed taking values γ times $\sigma_{T}$, where γ=2, 3, or 4 and $\sigma_{T}=1$.

Table 1 shows the theoretical values of the PIUR for various values of IUR, where the theoretical values are calculated based on the assumed distribution. For example, for a given value of IUR, the corresponding conditional probabilities for the FERE‐based *Z*‐scores can be computed as

$$Pr\left(Z_{FERE,i}^{\left(B\right)}>z_{p}\;|\;Z_{FERE,i}^{\left(A\right)}>z_{p},\;{\;\text{IUR}\;}_{i}=R\right)=\frac{\pi_{0}\Phi_{2,\rho }\left(-z_{p},-z_{p}\right)+\pi_{1}\Phi^{2}\left(s\right)}{\pi_{0}\Phi \left(-z_{p}\right)+\pi_{1}\Phi \left(s\right)},$$

where

$$s=-\frac{z_{p}}{\sqrt{1-\rho }}+\frac{\gamma }{\sqrt{2-2R}}.$$

The results shown in Table 1 suggest that, even when the IUR is small, relatively high PIUR can occur for settings including contamination with outliers. For example, even when the IUR = 0.00, if the proportions of outliers are set at 5% with the magnitude for these outlier provider effects taking values 2, 3, or 4 times $\sigma_{T}$, the corresponding FERE‐based PIURs are 0.56, 0.81, and 0.93, respectively.

**Table 1:** PIUR with various percentages of outliers

Outliers, %	IUR = 0.00			IUR = 0.25			IUR = 0.50		
	$2\sigma_{T}$	$3\sigma_{T}$	$4\sigma_{T}$	$2\sigma_{T}$	$3\sigma_{T}$	$4\sigma_{T}$	$2\sigma_{T}$	$3\sigma_{T}$	$4\sigma_{T}$
0		0.00			0.25			0.50	
1	0.27	0.55	0.71	0.41	0.64	0.77	0.57	0.75	0.83
2	0.39	0.73	0.83	0.49	0.79	0.87	0.62	0.83	0.90
5	0.56	0.81	0.93	0.61	0.86	0.94	0.70	0.91	0.97

*Note*: The magnitude for these outlier provider effects are fixed taking values γ times $\sigma_{T}$, where γ=2, 3, or 4; the results are based on *P* value of 0.025 using the FERE approach.

Abbreviations: FERE, fixed effects with random intercept; IUR, inter‐unit reliability; PIUR, profile inter‐unit reliability.

We next consider a linear model framework with 1000 providers and $n_{i}=100$ patients per provider. The continuous outcome, $Y_{ij}$, is generated from the linear model (2) with $\sigma_{w}^{2}=1$. The magnitude for these outlier provider effects are fixed taking values γ times $\sigma_{T}$, where γ=4. The remaining set ups are the same as those in Table 1. Table 2 shows that the empirical values of the profile IUR are close to the corresponding theoretical values.

**Table 2:** PIUR with various percentages of outliers

True IUR	Outliers, %	Total‐$\hat{\;\text{IUR}\;}$	EN‐$\hat{\;\text{IUR}\;}$	PIUR	FERE‐$\hat{\;\text{PIUR}\;}$	EN‐$\hat{\;\text{PIUR}\;}$
0.25	0	0.25	0.21	0.25	0.26	0.28
	1	0.35	0.23	0.77	0.79	0.76
	2	0.42	0.23	0.87	0.89	0.87
	5	0.57	0.25	0.94	0.96	0.94
0.50	0	0.50	0.47	0.50	0.51	0.52
	1	0.56	0.49	0.83	0.85	0.83
	2	0.61	0.49	0.90	0.93	0.90
	5	0.71	0.50	0.97	0.98	0.97

*Note*: Same sample size across providers; based on *P* value of .025.

Abbreviations: EN‐$\hat{IUR}$, estimated IUR based on the estimated null distribution; EN‐$\hat{PIUR}$, using the empirical null approach; FERE, fixed effects with random intercept; FERE‐$\hat{PIUR}$, using the FERE approach; IUR, inter‐unit reliability; PIUR, true PIUR; PIUR, profile inter‐unit reliability; Total‐$\hat{\;\text{IUR}\;}$, estimated IUR based on total between‐provider variation.

To assess the proposed methods in settings with various sample sizes across providers, we consider a linear model framework with 1000 providers and $n_{i}$ generated from a normal distribution with mean 100 and standard deviation 50 and then rounded to the nearest integer values. To avoid extremely small provider sizes, we set $n_{i}$ as 10 if the rounded integer value is less than 10. Tables 3 and 4 shows that the empirical values of the profile IUR are close to the corresponding theoretical values.

**Table 3:** PIUR with various percentages of outliers

True IUR	Outliers, %	Total‐$\hat{\;\text{IUR}\;}$	EN‐$\hat{\;\text{IUR}\;}$	PIUR	FERE‐$\hat{\;\text{PIUR}\;}$	EN‐$\hat{\;\text{PIUR}\;}$
0.25	0	0.24	0.22	0.25	0.23	0.29
	1	0.34	0.24	0.77	0.79	0.77
	2	0.41	0.25	0.87	0.89	0.86
	5	0.57	0.25	0.94	0.95	0.94
0.50	0	0.50	0.48	0.50	0.50	0.59
	1	0.56	0.49	0.83	0.84	0.83
	2	0.61	0.50	0.90	0.92	0.90
	5	0.71	0.49	0.97	0.98	0.97

*Note*: Various sample size across providers; based on *P* value of .025.

Abbreviations: EN‐$\hat{IUR}$, estimated IUR based on the estimated null distribution; EN‐$\hat{PIUR}$, using the empirical null approach; FERE, fixed effects with random intercept; FERE‐$\hat{PIUR}$, using the FERE approach; IUR, inter‐unit reliability; PIUR, true PIUR; PIUR, profile inter‐unit reliability; Total‐$\hat{\;\text{IUR}\;}$, estimated IUR based on total between‐provider variation.

**Table 4:** Estimated IUR and PIUR for SMR and SRR, with *P* value of .025; and using the empirical null approach

Measure	Year	$\hat{IUR}$	$\hat{\;P\text{IUR}\;}$	Number of facilities
SMR	2013	0.24	0.36	5424
	2014	0.25	0.39	5585
	2015	0.22	0.42	5770
	2016	0.23	0.38	5963
	2013‐2016	0.53	0.62	5965
SRR	2016	0.49	0.74	5740

Abbreviations: IUR, inter‐unit reliability; PIUR, profile inter‐unit reliability; SMR, standardized mortality ratio; SRR, standardized readmission ratio.

## 5 APPLICATION

In 2016, more than 120 000 patients were diagnosed with end‐stage renal disease (ESRD) (Saran *et al*., 2018), with kidney dialysis as the most common treatment option. In order to monitor the performance of dialysis facilities, several risk‐adjusted quality measures have been implemented by the Centers for Medicare and Medicaid Services (CMS) on the Dialysis Facility Compare (DFC) site and in the ESRD Quality Incentive Program, which is a CMS value‐based purchasing program. In this section, we apply the PIUR to two quality measures that are reported on the DFC site.

### 5.1 Standardized mortality ratio

The standardized mortality ratio (SMR) is a risk‐adjusted measure that is used to evaluate whether facility‐specific mortality rates are in line with the national average across all Medicare certified US dialysis facilities. The SMR for facility i is defined as ${\text{SMR}}_{i}=O_{i}∕E_{i}$, where $O_{i}$ is the observed number of deaths in facility i, and $E_{i}$ is the corresponding expected number of deaths for patients in this facility computed under a population norm. An SMR less (greater) than 1 indicates that the facility's observed death rate is less (greater) than expected based on overall national rates with adjustment for the measured characteristics of patients in this facility.

For practical implementation, the SMR is computed from a two‐stage model: in the first stage, a Cox model stratified by facilities is used to estimate regression parameters associated with patient characteristics. This model assumes that the hazard function is $\lambda_{ij}\left(t\right)=\lambda_{0i}\left(t\right)\text{exp}\left\{X_{ij}^{T}\beta \right\}$, where $\lambda_{0i}$ is the facility‐specific baseline hazard. This stratified approach avoids any problems that might arise when patient characteristics are correlated with facility effects. In the second stage, the regression parameters are used as an offset in an unstratified Cox model to estimate the baseline failure rate at the “average” facility. The expected number of events for the jth patient in the ith facility, denoted by $E_{ij}$, is calculated as $E_{ij}=\int_{0}^{\tau }R_{ij}\left(t\right)\text{exp}\left\{X_{ij}^{T}\hat{\beta }\right\}d{\hat{\Lambda }}_{0}\left(t\right)$, where τ is the maximal follow‐up time, $X_{ij}^{T}\hat{\beta }$ is treated as an offset with $\hat{\beta }$ estimated from stage 1, ${\hat{\Lambda }}_{0}\left(t\right)$ is the “population‐average” cumulative baseline hazard, and $R_{ij}\left(t\right)$ is the at‐risk process. The expected number for facility i is then computed as $E_{i}=\sum_{j=1}^{n_{i}}E_{ij}$. The corresponding *P* value can be computed using a Poisson approximation under which the $O_{i}$, under the null hypothesis that the facility's death rate is the same as the population average, follows a Poisson distribution with mean $E_{i}$.

**Figure 2:**
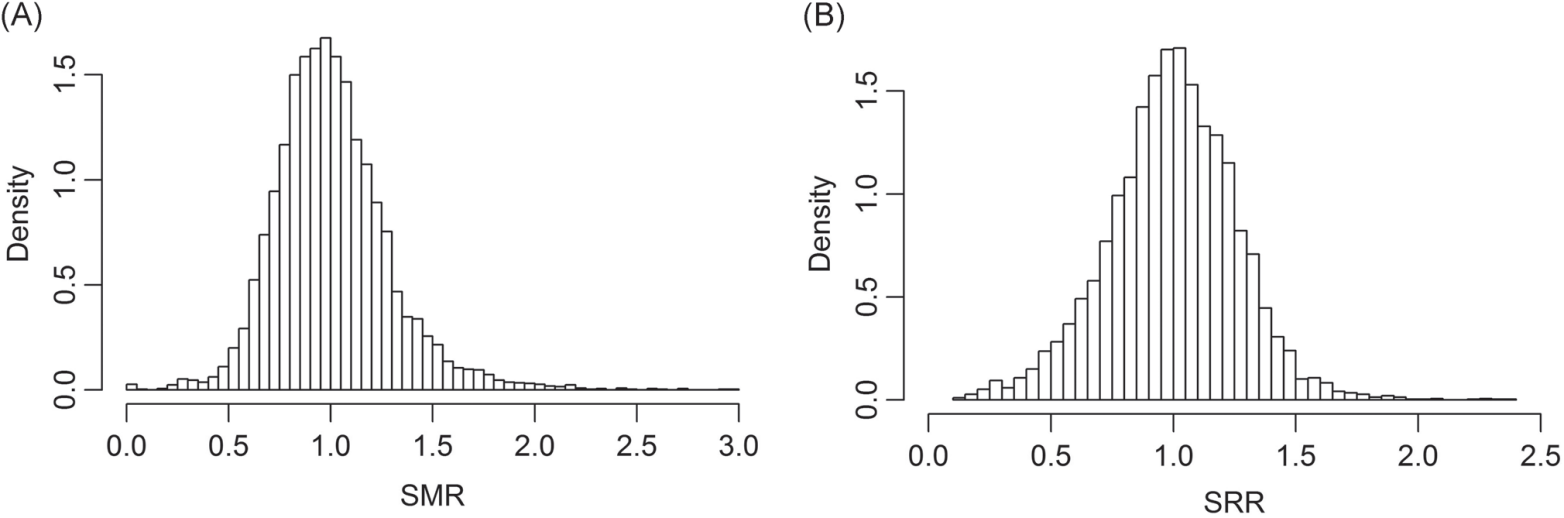
Histograms of SMR and SRR. A, The SMR figure is based on 5965 dialysis facilities with expected deaths greater than or equal to 3. B, The SRR figure is based on 5740 facilities with numbers of index discharges greater than 10. SMR, standardized mortality ratio; SRR, standardized readmission ratio

We use SMR data collected from the 4‐year period (2013‐2016). Methods of profiling are based on the empirical null approach. A total of 5965 facilities are included in the analysis, after excluding facilities with fewer than three expected deaths. The number of observed deaths per facility ranges from 0 to 418, and the number of expected deaths ranged from 3 to 309. Figure 2A shows a histogram of the SMR.

The IURs for SMR were computed based on the approach proposed by He *et al*. (2019). Based on 1‐year data, the IURs had a range of 0.22 to 0.25, which indicates that about one‐fourth of the variation in the SMR can be attributed to the between‐facility differences and about three‐fourths to within‐facility variation. Based on 4 years of data (2013 to 2016), the IUR for SMR increased to 0.53, which indicates that about half of the variation in the measure can be attributed to the between‐facility differences and about half to within‐facility variation. In comparison, with a *P* value of .025 and using the empirical null approach, the estimated conditional probability of being flagged again is ${\hat{\theta }}_{B|A}=0.22$, and the corresponding PIUR is $\tilde{R}=0.62$, which is computed based on the tabular theoretical values; for example, $G_{FERE}\left(\tilde{R}\right)=0.22$, where $G_{FERE}\left(\tilde{R}\right)$ is defined in Proposition 3. Note that in Table 1, when the IUR = 0.50, if the proportions of outliers are set at 2% with the magnitude for these outlier provider effects taking values two times $\sigma_{T}$, the corresponding PIUR is also 0.62. Thus, when there are outlier facilities, the PIUR tends to be larger than the IUR.

### 5.2 Standardized readmission ratio

An unplanned hospital readmission is defined as any unplanned hospital admission that occurs within 30 days of discharge from a previous admission. Readmissions are expensive. High readmission rates are indicators of poor care, leading to patient morbidity and poor quality of life, and can be prevented through effective post‐discharge early intervention and care coordination Chan *et al*. (2009). The standardized readmission ratio (SRR) is a measure of dialysis facility‐level hospital readmission among ESRD dialysis patients. It is computed as ${\text{SRR}}_{i}=O_{i}∕E_{i},$ where $O_{i}=\sum_{j=1}^{n_{i}}Y_{ij}$ is the number of observed readmissions, and $E_{i}=\sum_{j=1}^{n_{i}}E_{ij}$ is the expected number in facility i. Here $Y_{ij}$ is the observed outcome for the jth discharge in facility i, and $E_{ij}$ denotes the corresponding model‐based expected outcome, accounting for patient‐level characteristics and assuming the facility‐specific event rate equals the population rate. Specifically, readmission rates are modeled using a logistic model:

(4) $$\;\text{logit}\;\left(P_{ij}\right)=\log\left(P_{ij}∕\left(1-P_{ij}\right)\right)=\alpha_{i}+X_{ij}^{T}\beta ,$$

where $P_{ij}=P\left(Y_{ij}=1|\alpha_{i},X_{ij}\right)$, the parameters $\alpha_{i}$ correspond to the fixed facility effect and β is a vector of regression parameters. The expected number is computed as

$$E_{ij}=P_{ij}\left({\hat{\alpha }}_{M},\hat{\beta }\right)=\frac{\text{exp}\left({\hat{\alpha }}_{M}+X_{ij}^{T}\hat{\beta }\right)}{1+\text{exp}\left({\hat{\alpha }}_{M}+X_{ij}^{T}\hat{\beta }\right)},$$

where $\hat{\beta }$ is the estimate of β and ${\hat{\alpha }}_{M}$ denotes the median of all estimated facility effects. Similar to the interpretation of SMR, an SRR less (greater) than 1 indicates that the facility's observed readmission rate is less (more) than expected based on national rates.

Figure 2B shows the histogram of SRRs for 5740 dialysis facilities in 2016. The IUR had a value of 0.49, which indicates that about half of the variation in the SRR can be attributed to the between‐facility differences and about half to within‐facility variation. In contrast, with *P* value of 0.025 and using the empirical null approach, the profile IUR is 0.74, which indicates the existence of outlier facilities. For example, in Table 1, when the IUR = 0.50, if the proportions of outliers are set at 5% with the magnitude for these outlier provider effects taking values two times $\sigma_{T}$, the corresponding PIUR is 0.70. Thus, the difference between the PIUR and the IUR indicates the presence of outlier providers and, hence assesses more directly the ability of a quality measure for identifying outlier providers.

Note that although the IUR (0.53) for the SMR based on the 4 years of data is larger than the IUR (0.49) for the SRR based on 1 year of data, the difference between the PIUR and the IUR for SRR is larger than that for SMR. This indicates a larger proportion and/or higher magnitudes of outliers providers for SRR, which is not captured in the IUR itself.

## 6 DISCUSSION

The IUR is a metric that specifies the proportion of variation in the quality measure that is due to the between‐provider variation. If all between‐provider variation is due to quality of care and all patients and providers follow the assumed linear model, the IUR may be a reasonable signal to noise metric for a quality measure. However, in settings where the main source of between‐provider variation is due to incomplete risk adjustment or the main focus of the provider profiling is to identify outliers, the role of the IUR is limited. In fact, the IUR is based on the whole distribution of provider effects, and can be a poor indicator to assess the ability of a measure to identify outliers (Staggs and Cramer, 2016; Staggs, 2017; Kalbfleisch *et al*., 2018). Given that, the IUR may not be an appropriate metric for assessing the performance of a quality measure. In other words, the value of the IUR may not determine the suitability of a measure for identifying outliers; even measures with a small IUR can be very effective for identifying extreme providers, while a large IUR can be a signal of incomplete risk adjustment.

In this paper we assume that one main purpose of reporting quality measures is to reliably identify very good and very poor providers. To address the problems associated with the IUR, we propose an additional measure of reliability. The proposed PIUR is not designed to address the problem of “no unobserved confounders.” Instead, it is designed to assess the ability of quality measures to consistently identify outliers. Whether there are unobserved confounders or not, the values of the PIUR, compared with the IUR, are influenced by the proportion of outliers and their magnitude.

As proposed, the scale of the IUR is used to quantify the proposed PIUR, while the PIUR takes into account the providers with extreme outcomes. We have shown that the PIUR can be effective at assessing whether a quality measure reliably profiles outlier providers, even under low values of the IUR. If there are no outliers, one should expect the PIUR to be the same as the IUR. In cases where there are outlier providers, even measures with an IUR close to 0 can have relatively high PIUR and can be very useful for identifying extreme providers. Therefore, when the emphasis is on identifying “extreme” providers, the difference between the proposed PIUR and the IUR indicates the usefulness of the measure for profiling. In contrast, the size of the IUR or the PIUR value alone may not be a reasonable indicator of the use of a quality measure for identifying very good or poor providers. Therefore, we do not recommend a threshold for IUR or PIUR.

The values of the PIUR depend on the profiling method for flagging extreme providers. Both the commonly used FE and RE approaches assume that the provider effects are the consequence of variation in the quality of treatment and are under the full control of the providers. As a result, they will tend to identify as worse than expected, large providers, even when their true effect is not extreme. In contrast, the FERE approach is based on the assumption that most of the between‐provider variation is due to unobserved characteristics that are outside the control of the provider. Both of these extreme assumptions are typically invalid, and the provider effects correspond to a combination of quality of care and incomplete risk adjustment. Unfortunately, the source of the provider effect cannot be identified on the basis of the data alone and can only be estimated based on expert opinion. The article, Kalbfleisch *et al*. (2018) gives some discussion of how the various methods depend on the source of the variation. The empirical null approach has the advantage of leading to robust estimates of the between‐provider variance by limiting the influence of extreme observations. Additional discussion on this point can be found in Kalbfleisch and He (2018) and a working manuscript by Xia *et al*. (2019).

The covariates in our motivating settings are patient‐level, not provider level. However, a covariate may have both a between‐provider component, which we might summarize in terms of ${\bar{X}}_{i}$, the provider‐specific mean for provider i, and a within‐provider component $X_{ij}-{\bar{X}}_{i}$. This leads to differing within‐provider and between‐provider covariate effects as is discussed in Neuhaus and Kalbfleisch (1998). When provider effects are correlated with covariates, it should also be noted that use of a random effects model can yield a biased estimate of β (Pan, 2002; Kalbfleisch and Wolfe, 2013). The β that we are adjusting for is the within‐provider effect; one way to estimate that is to use a fixed effects model.

As illustrated in Section 5, the proposed method can also be applied to complex quality measures used in nonlinear models. Specifically, He *et al*. (2019) have developed methods to extend the IUR to such models. The empirical null approach described in Section 3.3 has been generalized to binary logistic model (He *et al*., 2013) and the Cox proportional hazards model (Kalbfleisch and Wolfe, 2013). Thus, the empirical null‐based PIUR can be easily computed to such nonlinear examples.

## Supporting information

Example R codes are available with this paper at the Biometrics website on Wiley Online Library.

Supplementary Information

Supplementary Information

Supplementary Information
