# Supplementary material for: The profile inter‐unit reliability
Source: Biometrics. 2019 Nov 10;76(2):654–63. doi: 10.1111/biom.13167 (PMC7318309; doi:10.1111/biom.13167)
Supplement: Supplementary file 1 — Supplementary Information [file BIOM-76-654-s001.pdf]

Supporting Information for "Designing Precision Medicine Trials to Yield Greater Population Impact" by

Ying-Qi Zhao*

and

Michael L. LeBlanc

Public Health Sciences Division, Fred Hutchinson Cancer Research Center

*Contact: Ying-Qi Zhao, yqzhao@fredhutch.org, Public Health Sciences Division, Fred Hutchinson Cancer Research Center, Seattle, WA 98109. The authors gratefully acknowledge support by R01DK108073, U10CA180819 and P30 CA015704 awarded by the National Institutes of Health and Coltman Early Career Fellowship awarded by Hope Foundation.

1 Additional details for examples in Section 2.1

Table 1: Model parameters

Example	a_{00}	a_{10}	a_{01}	a_{11}
1	0	0	0	0
2	0	0	0	2
3	0	0	-2	4
4	-3	4	-2	4

Figure 1: Non centrality curve and the ultimate impact curve of the enrichment clinical trial based on eligibility criterion $X > c$ for a screening size of 2000 ($ns = 2000$)

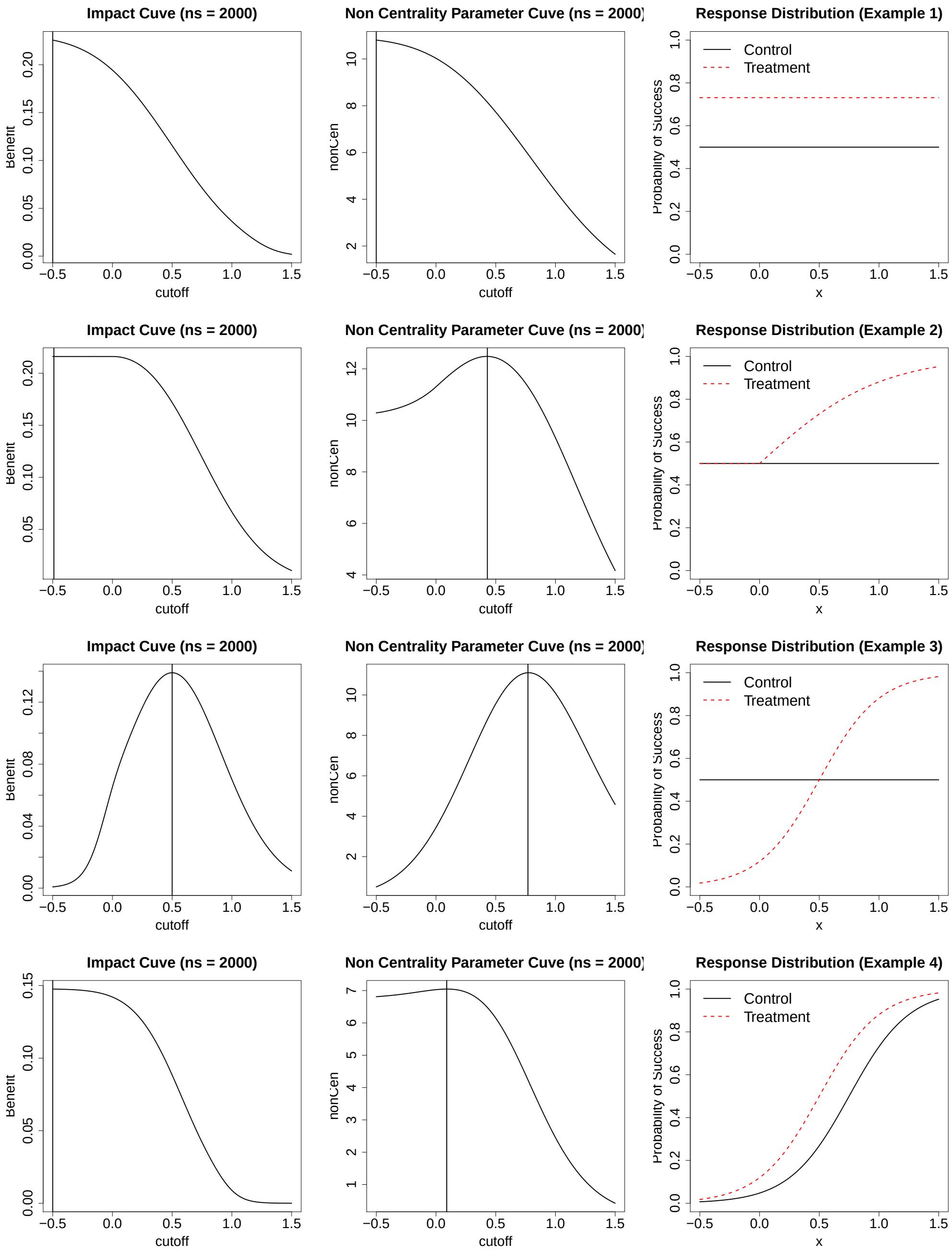

Figure 2: Non centrality curve and the ultimate impact curve of the enrichment clinical trial based on eligibility criterion $X > c$ for a screening size of 200 ($ns = 200$)

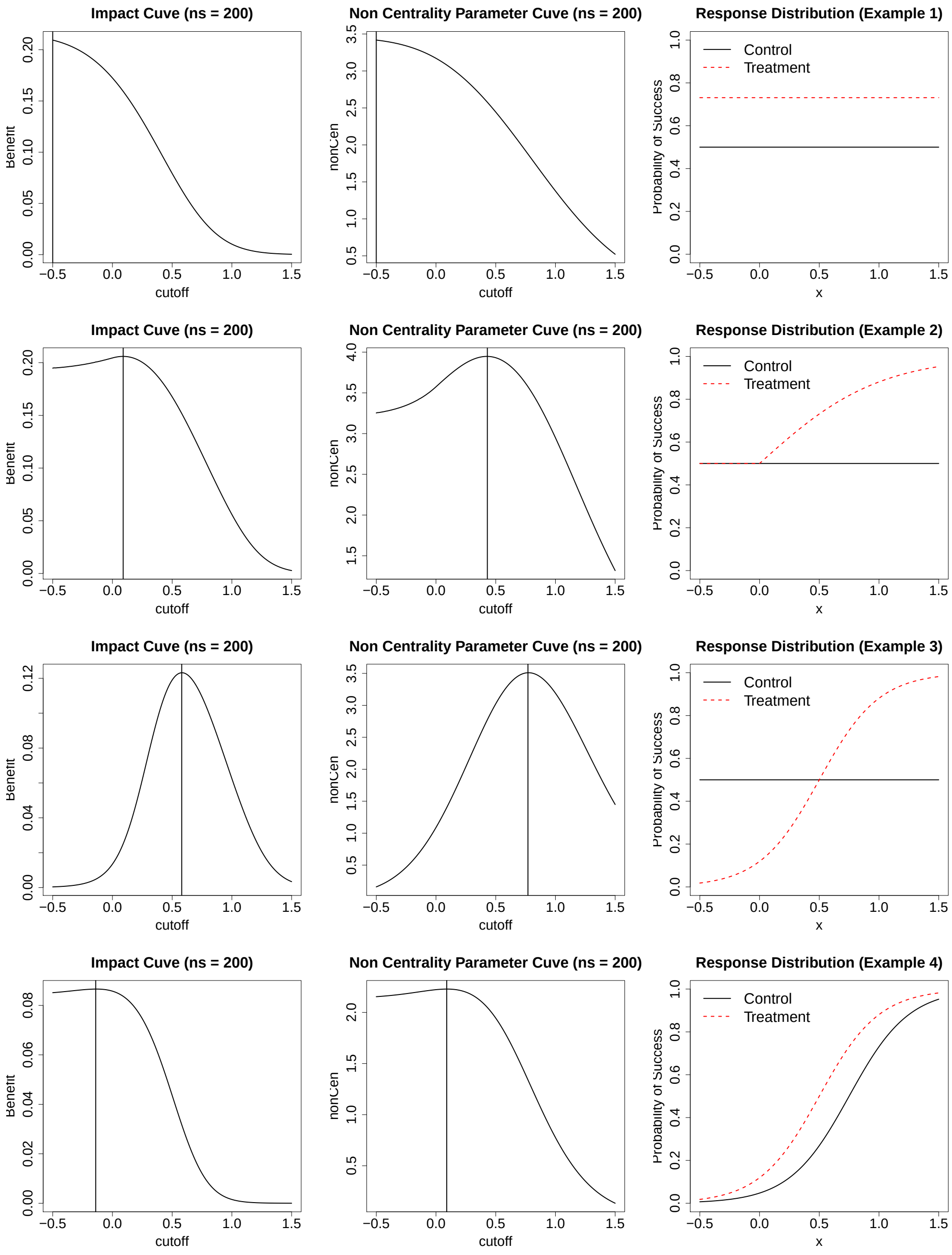

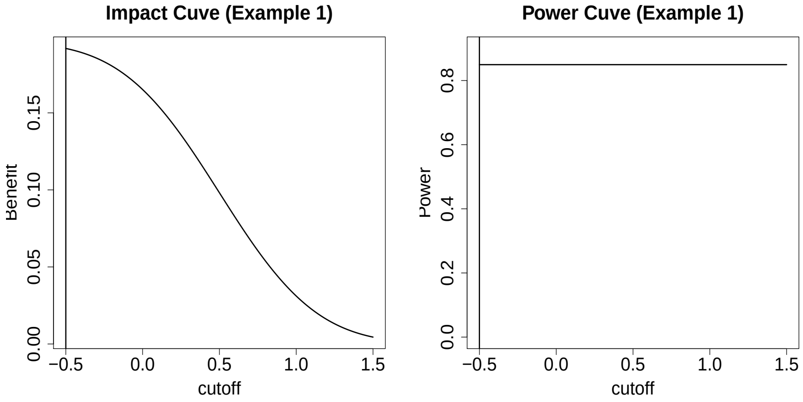

Figure 3: Power curve and the ultimate impact curve of the enrichment clinical trial based on eligibility criterion $X > c$ from Example 1 under fixed sample size for randomization

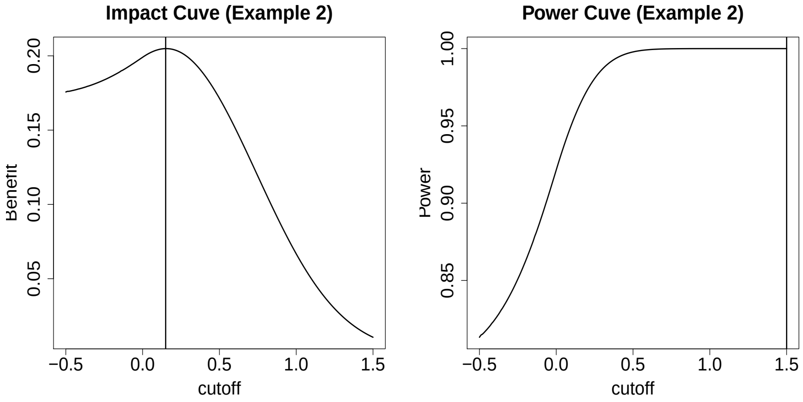

Figure 4: Power curve and the ultimate impact curve of the enrichment clinical trial based on eligibility criterion $X > c$ from Example 2 under fixed sample size for randomization

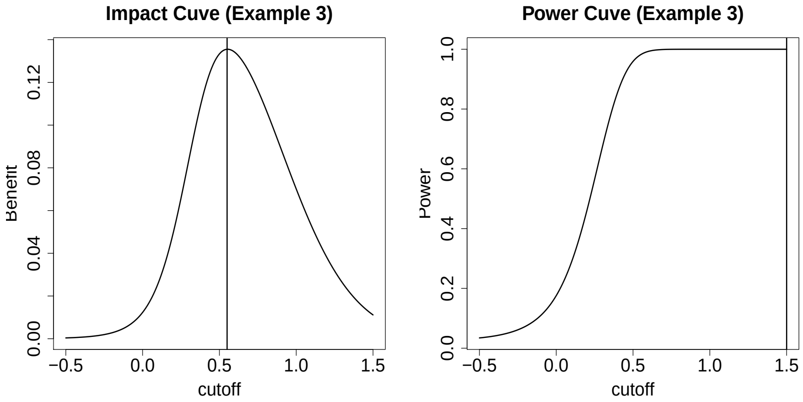

Figure 5: Power curve and the ultimate impact curve of the enrichment clinical trial based on eligibility criterion $X > c$ from Example 3 under fixed sample size for randomization

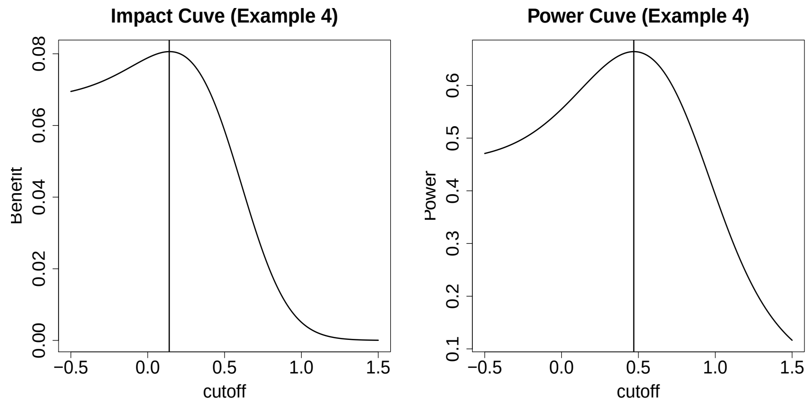

Figure 6: Power curve and the ultimate impact curve of the enrichment clinical trial based on eligibility criterion $X > c$ from Example 4 under fixed sample size for randomization
