# Supplementary material for: The profile inter‐unit reliability
Source: Biometrics. 2019 Nov 10;76(2):654–63. doi: 10.1111/biom.13167 (PMC7318309; doi:10.1111/biom.13167)
Supplement: Supplementary file 3 — Supplementary Information [file BIOM-76-654-s003.pdf]

Supporting Information

Web Appendices, Figures and simulation codes referenced in Sections 2 and 3 are available with this paper at the Biometrics website on Wiley Online Library.
